# Immunological combination treatment holds the key to improving survival in pancreatic cancer

**DOI:** 10.1007/s00432-020-03332-5

**Published:** 2020-08-03

**Authors:** M. H. Sodergren, N. Mangal, H. Wasan, A. Sadanandam, V. P. Balachandran, L. R. Jiao, N. Habib

**Affiliations:** 1 Department of Surgery and Cancer, Imperial College London, Hammersmith Campus, DuCane Road, London, W12 0HS UK; 2 Division of Molecular Pathology, Institute for Cancer Research, London, UK; 3 Centre for Molecular Pathology, Royal Marsden Hospital, London, UK; 4 Hepatopancreatobiliary Service, Department of Surgery, Memorial Sloan Kettering Cancer Center, New York, USA; 5 Parker Institute for Cancer Immunotherapy, Memorial Sloan Kettering Cancer Center, New York, USA; 6 David M. Rubenstein Center for Pancreatic Cancer Research, Memorial Sloan Kettering Cancer Center, New York, USA

**Keywords:** Combination, Immunotherapy, Pancreatic, Cancer

## Abstract

Advances in surgery, peri-operative care and systemic chemotherapy have not significantly improved the prognosis of pancreatic cancer for several decades. Early clinical trials of immunotherapy have yielded disappointing results proposing other means by which the tumour microenvironment serves to decrease the immune response. Additionally, the emergence of various subtypes of pancreatic cancer has emerged as a factor for treatment responses with immunogenic subtypes carrying a better prognosis. Herein we discuss the reasons for the poor response to checkpoint inhibitors and outline a rationale why combination treatments are likely to be most effective. We review the therapies which could provide optimal synergistic effects to immunotherapy including chemotherapy, agents targeting the stroma, co-stimulatory molecules, vaccinations and methods of immunogenic tumour priming including radiofrequency ablation. Finally, we discuss reasons why peri-operative and in particular neoadjuvant combination treatments are likely to be most effective and should be considered for early clinical trials.

## Introduction

Pancreatic ductal adenocarcinoma (PDAC) has a dismal prognosis and despite advances in surgical, anaesthetic and perioperative techniques, this has not significantly changed in the last half a century, and by 2030 PDAC is expected to become the second most common cause of death from cancer in the United States (Rahib et al. 2014). The high mortality is related to the proportion of patients that are diagnosed at a relatively late stage and in the 20% of patients who present with localised disease, the vast majority will develop recurrent disease following surgical resection. Although there have been encouraging non-randomised data relating to neoadjuvant FOLFIRINOX treatment, the fact is that PDAC is resistant to traditional chemotherapeutic agents currently available in clinical practice (Marsh et al. 2015). It is unlikely that significant further progress will be made in the technical surgical management of PDAC, as perioperative mortality is low and more aggressive resections (arterial, extended lymphadenectomy) have not significantly improved clinical outcomes (Jang et al. 2014; Jegatheeswaran et al. 2017). Even the small proportion of patients who are successfully downstaged to operable disease receive only a very modest overall survival advantage if any (Gillen et al. 2010; Suker et al. 2016). There is therefore an urgent need to address the systemic treatment of this aggressive disease.

### The era of immunotherapy

Until a decade ago, clinical outcomes from immunotherapy treatments such as interleukins and interferon were disappointing, however the more recent introduction of checkpoint inhibitors has generated a renewed enthusiasm in the field. Immune checkpoints are ligand-receptor interactions which can either stimulate or inhibit an immune response. The cytotoxic T-lymphocyte-associated protein 4 (CTLA-4) and the programmed cell death 1 (PD-1) proteins are inhibitory receptors present on activated T cells and are licensed immune checkpoint targets for some solid tumours (Wei et al. 2018). Inhibition of these receptor or ligands can augment the immune response of activated T cells against the tumour and has exhibited some remarkable clinical activity. CTLA-4 inhibition is instrumental in activation of suppressor regulatory T cells (Tregs) and is thought to exist primarily to prevent autoimmunity and excessive immune responses to infection (Johnson et al. 2017).

Nivolumab and pembrolizumab monotherapy (both monoclonal antibodies that block PD-1 from binding to its ligand) have resulted in long-term progression-free survival in a significant number of patients with metastatic melanoma. Furthermore, about 15% of patients treated with pembrolizumab achieved a complete response and of those 90% were disease-free at 2 years following discontinuation of treatment confirming a durable response and providing hope for a cure for many in this group (Robert et al. 2017). In the KEYNOTE-001 trial of patients with metastatic non-small cell lung cancer (NSCLC) who had progressed on platinum-based chemotherapy and expressed over 50% of PD-L1 in the tumour, the response rate to pembrolizumab was 45% (Garon et al. 2015). In 2017, pembrolizumab was licensed by the FDA for first line combination treatment of metastatic NSCLC and also for any solid tumours in both adults and paediatric patients that are microsatellite instability-high (MSI-H) or have a mismatch repair deficiency and that have progressed following prior treatment (Le et al. 2017). MSI-H or mismatch repair deficiency (MMR-d) occurs due to mutations that code for genes of mismatch proteins (MMR) proteins which recognize and correct errors in mismatched nucleotides or through methylation of the MLH1 promoter gene (Hu et al. 2018). These increased numbers of somatic mutations and hence MSI-H tumours express a great number of neoantigens (unique peptides that help immune cells identify and fight cancer cells) thereby making them more responsive to checkpoint blockade than tumours with few mutations (Macherla et al. 2018). In a small subset of PDAC cases, patients presenting with MSI-H tumours have responded to PD-1 blockade successfully (Eso et al. 2020). Although this group of patients is rare, it highlights the correlation between a greater mutational load and successful response to immune checkpoint therapy for PDAC. This could be translated to other subtypes of pancreatic cancer where the tumour could be primed with more mutations and in doing so would achieve the same beneficial response to immune checkpoint inhibitors.

Preliminary trials of checkpoint inhibitors in pancreatic cancer have yielded disappointing results. In a phase 2 trial of single agent ipilimumab (anti-CTLA-4) for locally advanced or metastatic pancreatic adenocarcinoma there was 1 delayed response in a treatment cohort of 27 patients (Royal et al. 2010). In a basket trial evaluating the safety and efficacy of an anti-PD-L1 antibody, none of the 14 patients with advanced pancreatic cancer experienced an objective response (Brahmer et al. 2012) however interim results of a multicentre, dose-expansion, phase I trial of the anti–PD-L1 monoclonal antibody durvalumab showed a disease control rate of 21% among 29 patients, 2 of whom achieved a partial response (Khalil and Segal 2016). Despite the results of these early trials, it is known that both CTLA-4 (in circulating CD8^+^ T cells) and PD-L1 expression are upregulated in a subset of PDACs and both are associated with worse survival (Farren et al. 2016; Zhang et al. 2017; Nomi et al. 2007). Furthermore, recently described gamma delta T (γδT) cells, which constitute up to 40% of tumour-infiltrating lymphocytes (TILs) in PDAC, have been found to express high levels of PD-L1 further contributing to suppression of T cell activation based on pre-clinical data (Daley et al. 2016). Based on these observations, one may therefore expect a better response to checkpoint inhibition in pancreatic cancer than what has been seen in early trials.

## Why does pancreatic cancer appear not to respond to checkpoint inhibitors in the same way as melanoma or NSCLC?

In order for the immune system to effectively attack cancer cells a process of recognition needs to take place which is initiated by antigen presenting cells (APC) that process tumour antigen onto the major histocompatibility complex (MHC) molecules located at the cell surface. This triggers expression of co-stimulatory molecules on the APC and migration to lymph nodes where the antigen is presented to T-cells through the antigen-specific T cell receptor (TCR). If the co-stimulatory molecules interact with their ligand on the T cell, then activation occurs, and the T cell leaves the lymph node. When an activated T cell recognises the tumour antigen, cytolytic enzymes and cytokines are released which induces proliferation, resulting in tumour lysis and the creation of memory T cells. A disruption at any point in this process can lead to tumour evasion from the immune system with most tumours encompassing unique and intrinsic mechanisms to avoid immune recognition. Melanoma and NSCLC, in part due to effect of UV radiation and smoking, respectively, harbour a great number of somatic mutations and therefore a high neoantigen load (tumour-specific peptides presented to MHC Class 1 molecules) which is thought to facilitate recognition by T cells activated by checkpoint blockade. For PDAC, the average mutational load is low as this cancer type is driven by recurrent copy number of alterations (Balli et al. 2017). However, encouraging evidence from long-term survivors of PDAC have indicated that the neoantigen quality, rather than the quantity may be modulating immunogenicity in this cancer, thus implying the neoantigenic repertoire to have been evolutionary selected in this cohort (Balachandran et al. 2017). They report in the tumour of long-term survivors (LTSs) both the presence of a high neoantigen number and an abundant CD8^+^ T-cell infiltrate. Specifically, these neoantigen qualities were characterised using a fitness model and were present in the tumour antigen MUC16 or CA125 (Balachandran et al. 2017). In a primary tumour with several clones, they observed that clones with high-quality neoantigens were lost in the metastatic samples in comparison to the clones with a low-quality neoantigen number. Therefore, they conclude clonal evolution bearing differences in immune fitness correlating with the quality of neoantigens present in the primary tumour and that neoantigens may be T-cell targets in LTSs (Balachandran et al. 2017). These findings support the notion that neoantigen quality modulates immunogenicity, clonal fitness and immunoselection during tumour evolution (Balachandran et al. 2017). It is therefore vital that we explore further the neoantigens that are associated with “quality” responses which could then be used as targets for immune checkpoint inhibitor therapy.

PDAC and other solid tumour types such as colorectal cancer with low responsiveness to single agent immunotherapy share some common traits consisting of a low number of tumour infiltrating lymphocytes (TILs) and a high number of T regulatory cells (Tregs) and myeloid-derived suppressor cells (MDSCs), which both serve to decrease the tumour-specific immune response (Martinez-Bosch et al. 2018). Furthermore, PDAC is associated with a characteristic stroma of dense desmoplasia comprising cancer-associated fibroblasts (CAFs), pancreatic stellate cells and extracellular matrix, which results in a tumour which is generally both hypoxic and hypovascular (Thomas and Radhakrishnan 2019). This very potent immunosuppressive tumour microenvironment (TME) is thought to exclude or restrict access of T cells from the tumour cells, a phenomenon known as immune privilege (Johnson et al. 2017) in which the tumour is protected from immune attack. It is therefore apparent that although theoretically checkpoint inhibition could provide a benefit it is likely that for PDAC, a combination treatment will be required to obtain satisfactory clinical efficacy.

## What other therapy or therapies provide optimal synergistic anti-tumour effect?

**Fig. 1:**
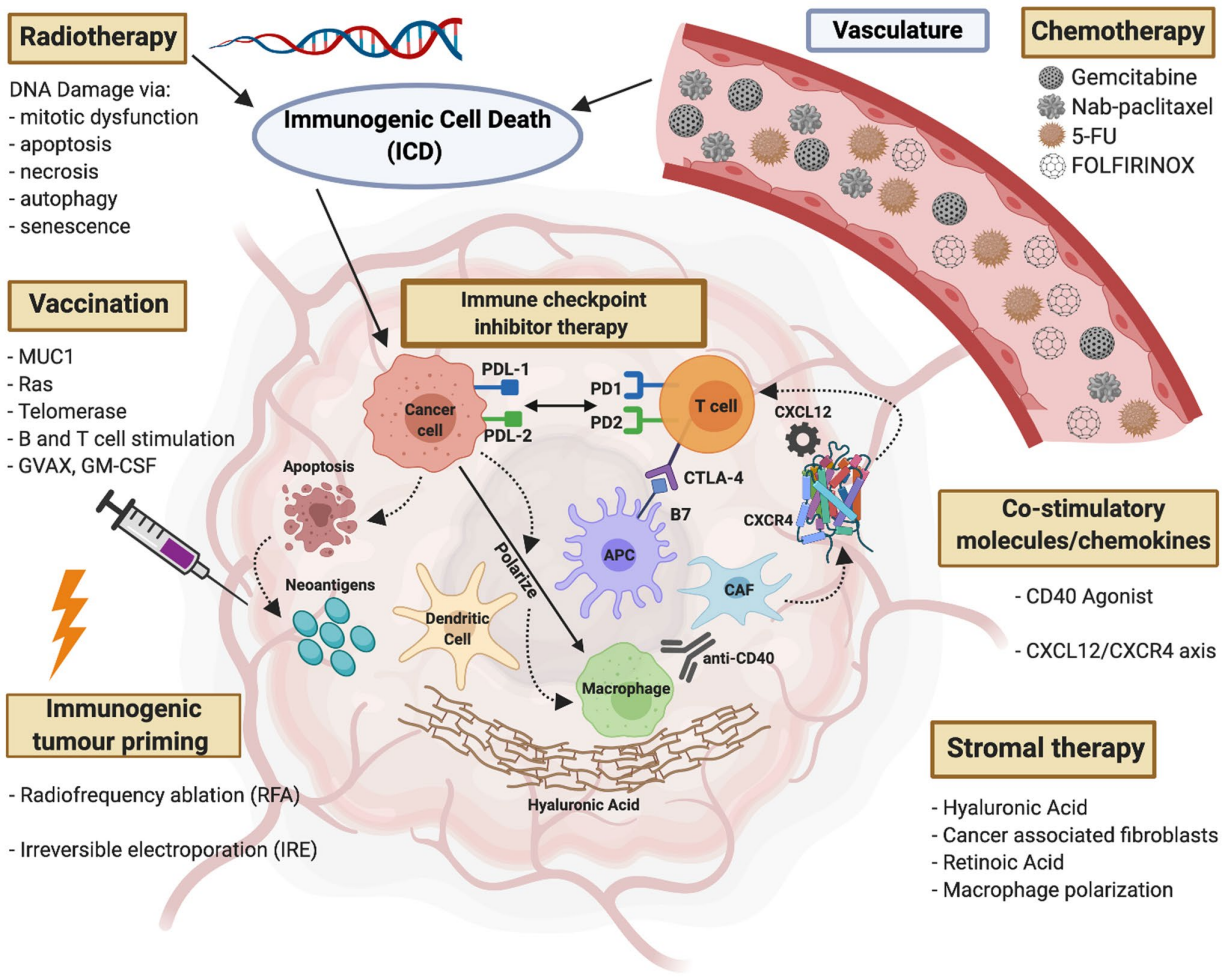
Summary diagram of the treatment methods of current and future use for combination therapy with immune checkpoint blockade. PDL-1 and PDL-2 receptors expressed on the surface of cancer cell, PD1, PD2 and CTLA-4 receptors expressed on T cells and B7 receptor on antigen presenting cell (APC). Combination therapies which include standard of care chemotherapy and radiotherapy, stromal targeting, co-stimulatory molecules/chemokines, vaccination, irreversible electroporation and radiofrequency aim to prime the tumour by overcoming mechanisms of resistance to immune checkpoint inhibitor therapy. Immunogenic cell death (ICD) caused by radiotherapy and chemotherapy causes the release of neoantigens. DNA damage via radiotherapy induces apoptosis, mitotic dysfunction, necrosis, autophagy and senescence of the cancer cells. Vaccination methods include but are not limited to direct targeting of tumour neoantigens such as MUC1, mutated protein Ras, telomerase, stimulation of B and T cells and GVAX which expresses GM-CSF. Radiofrequency ablation and irreversible electroporation prime the tumour by increasing antigen presentation and also enhance the activity of checkpoint blockade. Stromal therapy includes targeting Hyaluronic acid, Retinoic Acid and cancer-associated fibroblasts (CAF’s). Gemcitabine metabolism and pancreatic cancer cells have been reported to confer resistance intracellularly to therapies. Anti-CD40 in combination with gemcitabine and PD-1 blockade as a promising avenue to explore in reducing PDAC burden. Chemokine axis CXCL12/CXCR4 has been shown to contribute the immunosuppressive TME. In combination with PD-L1 treatment, an increase in T cell response has been observed rendering antitumor activity. Created with BioRender.com

In tumours exhibiting resistance to immune checkpoint inhibition, combinatorial approaches are emerging as a method to augment the response. These therapies include agents of standard of care chemotherapy and radiotherapy, stromal targeting, co-stimulatory molecules/chemokines, vaccination, irreversible electroporation and radiofrequency. These adjunct treatments aim to overcome the possible resistance mechanisms to sensitize the tumours to immune checkpoint blockade. Immunogenic cell death (ICD) caused by some cytostatic agents such as oxaliplatin and radiotherapy has been shown as a promising avenue for anti-cancer vaccines by release of tumour neoantigens (Young et al. 2018). Direct damage to the tumour releases more tumour peptides becoming available for processing by APC’s for subsequent T cell priming. In addition, the apoptosis of cancer cells results in the production of several factors that can be recognized by antigen presenting cells such as dendritic cells (DCs). Damage associated molecular patterns or DAMPs (endogenous molecules released upon stress or dying cells and carry a danger signal) released upon ICD may activate DCs to promote anti-tumour immunity by priming of naïve cytotoxic T cells via MHCII presentation, thus rendering as an in-situ vaccination and dendritic cell targeting (Galluzi et al. 2017; Green et al. 2009). Overview shown in Fig. 1.

### Chemotherapy

A combination with chemotherapy or using chemotherapy as a backbone for further combination treatment seems a reasonable place to start. Firstly, chemotherapy has known efficacy with a modest associated improvement in survival for PDAC and would likely be included as part of any first or second-line adjuvant clinical trial (Klaiber et al. 2018). Secondly, chemotherapies such as cyclophosphamide and gemcitabine are known to have suppressive effects on myeloid cells and Treg induction (Kan et al. 2012) leading to transient depletion and may therefore work in combination with checkpoint blockade to treat the immunosuppressive TME. The use of fluorouracil (5-FU), another chemotherapy agent has shown promising results in mice tumours to decrease myeloid-derived suppressor cells (MDSC) and increase IFN-ƴ production by tumour specific CD8^+^ T cells infiltrating the tumour, promoting T cell-dependent anti-tumour responses in vivo (Vincent et al. 2010).

Clinical trials exploring the combination of chemotherapy agents with immune checkpoint inhibitors CTLA-4 and PD-1 have shown interesting data in unresectable, locally advanced and metastatic cases of pancreatic cancer but remain low for resectable cases (Nomi et al. 2007; Kamath et al. 2019; Thind et al. 2017). Nomi et al., investigated the combination of pidilizumab and gemcitabine for resected pancreatic cancer and pembrolizumab and FOLFIRINOX for advanced GI cancer and found in vivo an increase in tumour infiltration of CD8^+^ T cells and a complete response (Nomi et al. 2007). There have also been other immune pathway targets explored in combination with chemotherapy in reducing PDAC burden. One study has reported in vitro inhibition of the receptor tyrosine kinase Axl which is associated with immune evasion and drug resistance in PDAC to have enhanced gemcitabine efficacy in vivo (Ludwig et al. 2017). Ma et al. report a significantly longer overall survival in the combination of immune checkpoint inhibitors and chemotherapy than chemotherapy alone in advanced pancreatic cancer (18.1 vs. 6.1 months) (Ma et al. 2020).

Perioperative chemotherapy has gained interest over the recent years in patients who present with localised and specifically resectable pancreatic cancer. Although it is role as a possible standard of care treatment for PDAC remains under debate because of the lack of stout data, there are several advantages associated. Firstly, to treat micrometastatic disease early to avoid delays of therapy and reduce the burden of unpredictable postoperative recovery. Secondly, to achieve negative surgical margins (R0 resection: microscopically complete resection of the tumour) by downsizing the tumour. Thirdly to study and determine the biological behaviour of the individual’s tumour following chemotherapy and resection (Demir et al. 2015). In other gastro-intestinal cancers such as oesophageal cancer, the use of neoadjuvant chemoradiotherapy is the standard of care treatment for resectable tumours and has been shown to improve disease-free survival, overall survival, R0 resection rate and pathological complete response (Hagen et al. 2012; Oppedijk et al. 2014; Shapiro et al. 2015).

SWOG S1505 is a randomized phase II study of patients with resectable disease aimed at determining the most promising perioperative regimen to test in a larger trial. Patients received 12 weeks of neoadjuvant chemotherapy with either modified FOLFIRINOX or gemcitabine/nab-paclitaxel followed by surgical resection in one arm and 12 weeks of postoperative chemotherapy in the other. However, we are still awaiting clinical outcomes to be reported later this year (NCT02562716) (Sohal et al. 2017). Additional recruiting trials include NEOPAC study (NCT01314027, neoadjuvant gemcitabine/oxaliplatin plus adjuvant gemcitabine vs. adjuvant gemcitabine in resectable pancreatic cancer), however this study was eliminated due to low recruitment (Heinrich et al. 2011), the NEPAFOX trial (NCT02172976, neoadjuvant/ adjuvant FOLFIRINOX vs. adjuvant gemcitabine in resectable pancreatic cancer) (Hozaeel et al. 2015). NEONAX-trial is at the moment the largest neoadjuvant trial with an extended translational program in resectable pancreatic cancer worldwide and has started recruitment in Q1/ 2015 at 26 German university hospitals for PDAC surgery (Ettrich et al. 2018). Table 1 lists current ongoing clinical trials of peri-operative, adjuvant and combination therapies for resectable, borderline resectable and locally advanced PDAC, obtained from clinicaltrials.gov.

**Table 1:** Ongoing clinical trials for neoadjuvant, adjuvant and combination with immunotherapy for resectable, borderline resectable and locally advanced pancreatic adenocarcinoma (www.clinicaltrials.gov)

Trial name and ID	Patient’s resectability	Phase	*n*	Neoadjuvant	Adjuvant	Combination with immunotherapy	Status	NCT no
PANACHE01: Neo-adjuvant FOLF(IRIN)OX for resectable pancreatic adenocarcinoma	rPDAC	II	160	+	−	−	Recruiting	NCT02959879
CISPD-1: sequential use of Gem/nab-P and mFOLFIRINOX as neoadjuvant CTX	rPDAC	II	416	+	−	−	Recruiting	NCT03750669
NEPAFOX: randomized multicentre phase ii/iii study with adjuvant gemcitabine versus neoadjuvant/adjuvant folfirinox for resectable pancreas carcinoma	rPDAC	II/III	40	+	+	−	Active, not recruiting	NCT02172976
NEONAX: neoadjuvant plus adjuvant or only adjuvant Gem/nab-P (17)	rPDAC	II	166	+	+	−	Active, not recruiting	NCT02047513
SWOG 1505: perioperative mFOLFIRINOX vs. Gem/nab-P	rPDAC	II	112	+	−	−	Active, not recruiting	NCT02562716
Neoadjuvant/adjuvant GVAX pancreas vaccine (With CY) with or without nivolumab and urelumab trial for surgically resectable pancreatic cancer	rPDAC	I/II	62	+	+	−	Recruiting	NCT02451982
Perioperative therapy for resectable and borderline-resectable pancreatic adenocarcinoma with molecular correlates	rPDAC and brPDAC	II	50	+	−	−	Recruiting	NCT02723331
nITRo: nal-IRI/5-FU/LV and oxaliplatin	rPDAC	II	67	+	−	−	Recruiting	NCT03528785
Nivolumab in combination with chemotherapy before surgery in treating patients with borderline resectable pancreatic cancer	brPDAC	I/II	36	+	−	−	Recruiting	NCT03970252
Study of pembrolizumab with or without defactinib following chemotherapy as a neoadjuvant and adjuvant treatment for resectable pancreatic ductal adenocarcinoma	rPDAC	II	36	+	+	+	Recruiting	NCT03727880
Alternative neoadjuvant chemotherapy in resectable and borderline resectable pancreatic cancer	rPDAC and brPDAC	I	30	+	−	−	Recruiting	NCT03703063
Nalirinox neo-pancreas RAS Mut ctDNA study	rPDAC	II	20	+	−	−	Recruiting	NCT04010552
Testing the use of the usual chemotherapy before and after surgery for removable pancreatic cancer	rPDAC	III	352	+	−	−	Not yet recruiting	NCT04340141
Study evaluating neoadjuvant immunotherapy in resectable pancreatic ductal adenocarcinoma	rPDAC	II	40	+	+	+	Recruiting	NCT03979066
Study of NAC of GA therapy for patients with BRPC	brPDAC	II	60	+	−	−	Recruiting	NCT02926183
PRIMUS002: looking at two neo-adjuvant treatment regimens for resectable and borderline resectable pancreatic cancer	rPDAC and brPDAC	II	278	+	−	−	Recruiting	NCT04176952
Pre-operative treatment for patients with untreated pancreatic cancer	rPDAC and brPDAC	II	24	+	−	−	Recruiting	NCT03138720
Testing the combination of two approved chemotherapy drugs and radiation prior to surgery in localized pancreatic cancer	LAPC	II	30	+	+	−	Recruiting	NCT03492671
Trial of neoadjuvant and adjuvant nivolumab and bms-813160 with or without gvax for locally advanced pancreatic ductal adenocarcinomas	LAPC	I/II	30	+	+	+	Recruiting	NCT03767582
Pooled mutant KRAS-targeted long peptide vaccine combined with nivolumab and ipilimumab for patients with resected MMR-p colorectal and pancreatic cancer	MMR-p PDAC	I	30	−	+	+	Not yet recruiting	NCT04117087
VX15/2503 and immunotherapy in resectable pancreatic and colorectal cancer	rPDAC	I	32	+	−	+	Recruiting	NCT03373188
BMS-813160 with nivolumab and gemcitabine and nab-paclitaxel in borderline resectable and locally advanced pancreatic ductal adenocarcinoma (PDAC)	brPDAC and LAPC	I/II	53	+	−	+	Recruiting	NCT03496662

*PDAC* pancreatic ductal adenocarcinoma, *rPDAC* resectable PDAC, *brPDAC* borderline resectable PDAC, *LAPC* locally advanced PDAC, *MMR-p* mismatch repair-proficient, *Gem/nab-P* gemcitabine/nab-paclitaxel, *NAC* neoadjuvant gemcitabine/nab-paclitaxel

Encouraging data culminated so far from both clinical trials and in vivo studies should be used to design trials investigating most efficacious combinations of chemotherapy and immunotherapy targets in perioperative settings per se. Table 1 lists current ongoing clinical trials of peri-operative, adjuvant and combination with immunotherapy for resectable, borderline resectable and locally advanced PDAC, obtained from clinicaltrials.gov. We have summarised a few of the studies evaluating the combinatorial approach thus far. Clinical trial NCT03727880 aims to evaluate whether the reprogramming of the TME through targeting focal adhesion kinase (FAK) post chemotherapy can potentiate PD-1 antibody before and after surgery. NCT03979066 aims to determine if administration of the monoclonal antibody; atezolizumab and PEGPH20 treatment before and after surgery, followed by chemotherapy is safely tolerated and if there is an increase in the immune response against the tumour rather than increase the chance of cure. Study NCT03767582′s purpose is to evaluate if the combination of nivolumab and a CCR2/CCR5 dual antagonist (BMS-813160) with GVAX is safe in patients with locally advanced pancreatic cancer (LAPC) who have received chemotherapy and radiotherapy and to also evaluate if this combination therapy enhances the infiltration of CD8^+^CD137^+^ cells in PDAC. NCT04117087 is a phase 1 study investigating resected PDAC and mismatch repair (MMR)-p colorectal cancer (CRC) to evaluate safety and the immune response to pooled mutant-KRAS peptide vaccine (KRAS peptide vaccine) with poly-ICLC adjuvant in combination with nivolumab and ipilimumab after adjuvant standard of care treatment. Clinical study, NCT03373188 is a randomized phase I trial evaluating the efficacy of anti-semaphorin 4D (anti-SEMA4D) monoclonal antibody VX15/2503 with and without ipilimumab or nivolumab in treating patients with stage I-III pancreatic cancer that is resectable or locally advanced. Study NCT03496662 aims to test treatment efficacy of BMS-813160 (CCR2/5 inhibitor), nivolumab, gemcitabine, and nab-paclitaxel in borderline and locally advanced PDAC.

### Radiotherapy

Alongside its direct cytotoxic effect in solid cancers, radiation therapy (RT) has been shown to modify the immunosuppressive tumour microenvironment of PDAC (Young et al. 2018). Radiation enhances anti-tumour activity through DNA damage leading to mitotic dysfunction, apoptosis, necrosis, autophagy and senescence (Liu et al. 2018). Emerging preclinical data suggest that radiation in combination with PD-1/PDL-1 blockade confers immune-stimulatory effects (Azad et al. 2017). There are currently many ongoing clinical studies underway investigating RT with single/dual checkpoint blockade in pancreatic cancer patients, these are in early stages as parameters such as dosing and fractionation of the therapy will need to be established for optimal clinical benefit (Young et al. 2018; Liu et al. 2018; Azad et al. 2017).

### Stromal targeted therapy

The PDAC TME stroma can be targeted in several ways. Hyaluronic acid (HA) is a glycosaminoglycan commonly found in the extracellular matrix (ECM) of PDAC and contributes to the physical barrier associated with treatment resistance. It also binds to cell surface receptors which stimulate tumorigenesis. PEGPH20 is an agent that targets the accumulation of HA within the TME and has shown some promising in vivo and pre-clinical results (Thompson et al. 2010; Provenzano et al. 2012) and a phase 2 randomised controlled trial showed a limited improvement in disease-free survival in combination with chemotherapy for treatment naïve stage 4 disease (Hingorani et al. 2018), however ongoing phase 3 data are awaited. The Sonic hedgehog (Shh) signalling pathway has been shown to be overexpressed in PDAC cells and also illustrate aberrant activation of CAFs promoting a desmoplastic TME. Shh inhibitors are thought to enhance delivery of chemotherapy through depletion of stromal tissue and increase in vascular density although promising pre-clinical data (Olive et al. 2009) have not been replicated in clinical trials (Catenacci et al. 2015). A further example of stromal co-targeting is with all-trans retinoic acid, thought to dampen multiple signalling cascades in the tumour-stroma crosstalk, which has yielded some encouraging pre-clinical results combined with gemcitabine (Carapuca et al. 2016). The metabolic effect of the stroma on immune response is critical for paving the way to understanding why chemotherapy fails in some patients. Increasing evidence from many studies have shown the stroma to confer intrinsic resistance to gemcitabine by modifying the genes involved in gemcitabine metabolism and activating intracellular antiapoptotic signalling pathways (Dangi-Garimella et al. 2013). In addition, Halbrook et al., have recently shown pancreatic cancer cells to polarize macrophages and release pyrimidine nucleosides such as deoxycytidine which directly competes with gemcitabine (Hallbrook et al. 2019). CAFs make up the bulk of this desmoplastic stroma of PDAC. The majority of pancreatic stellate cells (PSC) are CAFs in PDAC and store vitamin A-containing lipid droplets in their quiescent state. When activated PSCs lose their storage function, ECM proteins and pro-tumoural factors (Neuzillet et al. 2019). Many emerging studies have shown CAFs to bridge the gaps in the heterogeneous phenotypes of PDAC (Hallbrook et al. 2019; Neuzillet et al. 2019; Öhlund et al. 2017). It has therefore become the topic of interest for many, as deciphering this interplay between these cells may provide not only insights into the heterogeneous makeup of PDAC but may prove as a powerful tool to navigate patient-specific treatment. Additionally, they have also been reported to be involved in drug resistance, which makes them an imperative target (Neuzillet et al. 2019).

**Fig. 2:**
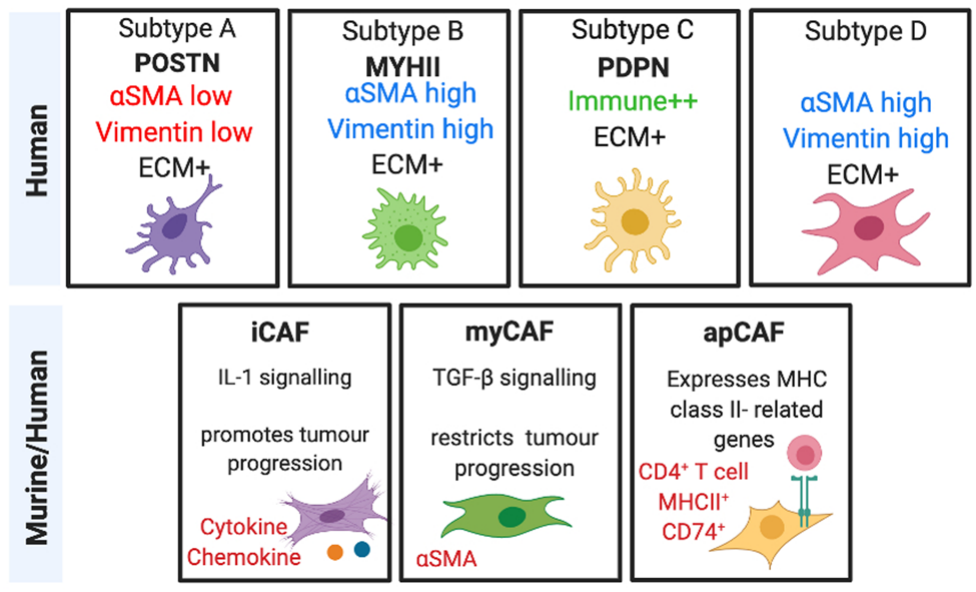
Cancer-associated fibroblast (CAF) subtypes from murine and human analysis studies. Top panel represents subtypes A-D of CAF characterised from patient-derived samples with distinct molecular features and prognosis impact. Subtypes A, B and D classify under the poor/intermediate prognosis whereas subtype C has a good prognosis (Neuzillet et al. 2019). Bottom panel represents the major CAF types culminated from both murine and human analysis (Elyada et al. 2019). Created with BioRender.com

**Fig. 3:**
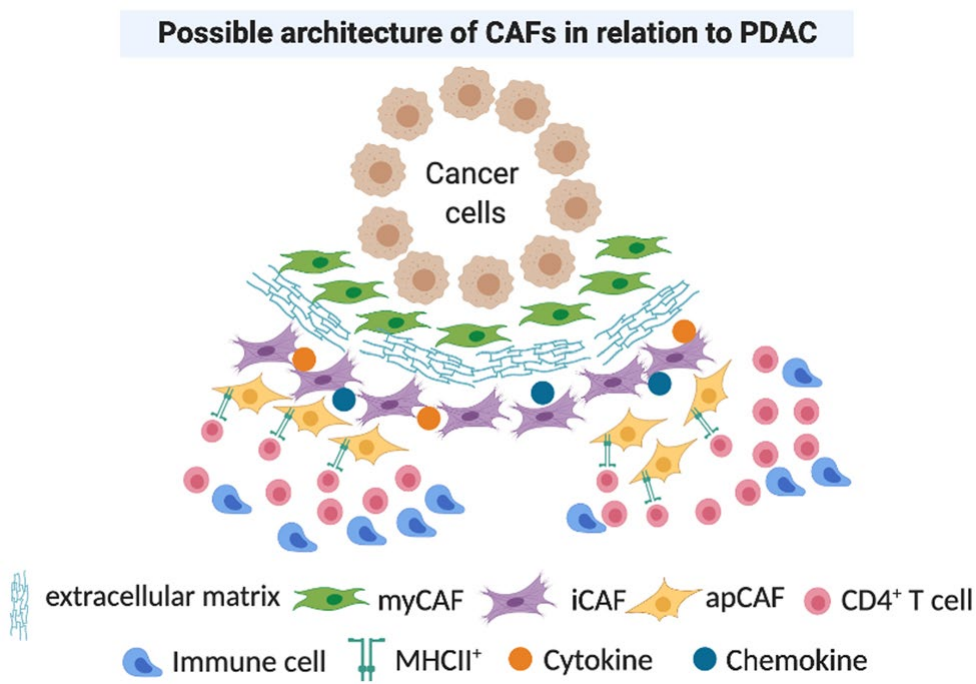
The possible configuration of the cancer-associated fibroblasts (CAFs) spatial relation in PDAC. Myofibroblastic CAFs (myCAF) surround the tumour closely whereas the inflammatory CAF (iCAF) and antigen presenting CAFs (apCAF) locate more distally from the tumour but in close proximity to immune cells (Elyada et al. 2019). Created with BioRender.com

Single cell analysis studies report the heterogeneity observed within these populations of CAFs and classify them according to their unique characteristics (Neuzillet et al. 2019; Öhlund et al. 2017). To understand the level of heterogeneity observed, it is important to take note of the differences in both mouse models and those from patient resected samples. To date there has been two major CAF subtypes reported which includes myofibroblastic CAFs (myCAFs), these express a high level of α-smooth muscle actin (αSMA) and reside closely to the tumour and inflammatory CAFs (iCAFs) which express lower levels of αSMA but high levels of chemokines and cytokines and locate more distally from the tumour (Öhlund et al. 2017). Recently a third subtype of CAF has been identified and this subset which they classify as antigen-presenting CAFs (apCAFs) expresses MHC class II-related genes to CD4^+^ T cells and modulates the immune axis capacity (Neuzillet et al. 2019) (Figs. 2 and 3). This provides as an interesting avenue to explore because if the apCAFs are inhibiting the activity of T cells, then a therapy to target this CAF would be critical and in combination with complementary immune checkpoint blockade could revolutionize personalised medicine (Öhlund et al. 2017). Furthermore, CAFs have been shown to shift between varying identities and this plasticity could potentially be exploited to render efficient therapeutic responses whereby the paradigm is tilted towards an anti-tumorigenic CAF rather than a pro-tumorigenic one (Neuzillet et al. 2019). This transitory observation in CAFs could also explain why there are inconsistences observed in preclinical trials targeting the stroma (Elyada et al. 2019). In contrast, Neuzillet et al. report four subtypes to exist based on analysis of patient resected samples, each represented by differences in molecular and functional features which include ECM, immune signatures, intra-tumoural spatial pattern of expression of vimentin, αSMA and proliferation rate, tumour promoting and chemoprotective capabilities and distinct prognostic impact (Elyada et al. 2019) (Figs. 2 and 3). These CAF subtypes were also found to co-exist in multiples within a tumour sample, indicating towards the inconsistencies seen in clinical trials that specifically target the stroma (Elyada et al. 2019).

### Co-stimulatory molecules/chemokines

Another interesting target is the CD40 co-stimulatory protein found on macrophages and dendritic cells, required for activation by CD4^+^ T helper cells, and needed to transform CD8^+^ T cells into cytotoxic effector T cells. Activation of CD40 by monoclonal antibodies in combination with gemcitabine has shown a 19% response rate in patients with advanced PDAC (Beatty et al. 2013). The Parker Institute for Cancer Immunotherapy are currently recruiting to a multi-centre phase 1b/2 trial evaluating the safety and efficacy of CD40 agonistic monoclonal antibody (APX005M) administered together with gemcitabine and nab-paclitaxel with or without PD-1 blocking antibody (nivolumab) in patients with previously untreated metastatic pancreatic adenocarcinoma (clinicaltrials.gov NCT03214250). A Phase 1 study evaluating the effect of anti-CD40 in the neoadjuvant setting for resectable PDAC is also currently recruiting (clinicaltrials.gov NCT02588443).

The chemokine CXCL12/CXCR4 axis leads downstream to CAFs expressing fibroblast activation protein and further contributing to the immunosuppressive TME in PDAC. CXCR4 inhibition has been shown in preclinical models combined with PD-L1 treatment to induce a robust T cell response and to have antitumor activity (Feig et al. 2013). The chemokine-receptor CCL2/CCR2 signalling axis has also been targeted in PDAC and in preclinical models has led to depletion of inflammatory monocytes and macrophages resulting in an enhanced T cell response and inhibition of tumour growth in pre-clinical studies (Mitchem et al. 2013) and a 48% response rate in combination with FOLFIRINOX chemotherapy (baseline ~25%) in patients with borderline or locally advanced PDAC, although the size of the cohorts were small for both groups, the data represents an interesting avenue to explore in combining chemotherapy with targeted pathways in PDAC (Nywening et al. 2016).

### Vaccination

Much interest has been generated by the idea of producing a PDAC cancer vaccination and how such a therapy can work in combination with others. A vaccination would aim to stimulate the immune system through enhanced presentation of tumour-specific antigens and thereby produce a B and T cell response. It is not possible to summarise all cell-based, peptide or genetic approaches here however common targets have included human MUC1 protein, mutated Ras protein and telomerase (Paniccia et al. 2015). GVAX is a whole cell vaccine composed of two irradiated pancreatic cancer cell lines engineered to express granulocyte–macrophage colony-stimulating factor (GM-CSF) with mesothelin as one of the preferred antigenic targets. Phase 2 adjuvant studies combining GVAX with chemoradiotherapy indicated a favourable clinical response (Lutz et al. 2011). An interesting phase 1 study evaluating safety of an anti-CTLA-4 (Ipilimumab) alone or in combination with GVAX in heavily pre-treated patients showed an immune response induction and clinical activity after prolonged treatment, but also a significantly increased overall survival in combination versus monotherapy (Le et al. 2013).

### Immunogenic tumour priming

Another very interesting method to increase activity of checkpoint blockade is combination with a method of tumour tissue destruction that increases antigen presentation to the immune system. Examples include stereotactic radiotherapy, cryoablation and radiofrequency ablation (RFA). RFA induces thermal coagulative necrosis through local application of radiofrequency waves, leading to irreversible destruction of the tumour. The localised coagulation necrosis of the tumour remains in the body and is thought to provide proinflammatory signals and induce the release of large amounts of cellular debris that represents a source of tumour antigens that can trigger a host adaptive immune response against the tumour. This results as a pathogenic “noxa” (exerting a harmful effect) for the body, inducing a strong inflammatory response (Evrard et al. 2007). Indeed, in the mouse model, it has been observed that tumours partially treated with RFA do not only exhibit this response against the primary tumour, but they also observed an abscopal effect, i.e., regression of non-ablated or distant tumour sites owing to induction of T cell responses. Furthermore, the animals who exhibited irradiation of the primary tumour were able to resist a further challenge with tumour implant, implying a durable immunological memory response (Dromi et al. 2009). Interestingly, in the neoadjuvant setting in a murine model, improved survival and antitumour systemic immunity was seen in pre-resectional RFA treatment, supporting its use in cancers associated with a high risk of local or systemic recurrence (Ito et al. 2015). It has been demonstrated in advanced human cancers that RFA ablation induces an increased tumour-associated antigen-specific antibody reactivity (Widenmeyer et al. 2011) although the response is likely clinically weak in advanced disease and not sufficient for complete eradication of established tumours. Nevertheless, in a clinical trial of non-small cell lung cancer (NSCLC) Schneider and colleagues demonstrated for the first time a local and systemic immune response subsequent to RFA and then complete surgical resection in patients. Along the perimeter of the RFA-treated tumour tissue they found intense infiltrations of CD4^+^ and CD8^+^ lymphocytes following resection. In the peripheral blood, the frequency of proinflammatory and immunostimulatory dendritic cells increased after RFA and in T-cell assays a significant increase in T-cell proliferation was detected after RFA and tumour resection. They concluded that the treatment of NSCLC patients with RFA and surgery leads to an activated and highly T-cell-stimulatory phenotype of dendritic cell and that this activation may promote long-term immunity (Schneider et al. 2016). Shi et al. have found that RFA treatment in colorectal cancer patients not only increased T cell infiltration, but also PD-L1 expression of the tumour (Shi et al. 2016). This provides a mechanism by which the tumour can evade the immune response and a potential target for combination therapy. Indeed, combining RFA with a CTLA-4 inhibitor has demonstrated clinical activity in a phase 1 trial of hepatocellular carcinoma with a clear increase in CD8^+^ T cells in patients showing a clinical benefit only (Duffy et al. 2017). In a study of patients undergoing RFA for locally advanced pancreatic cancer, Giardino and colleagues showed that CD4^+^, CD8^+^ and effector memory T cells increased from day 3 and myeloid antigen presenting dendritic cells increased at day 30, suggesting activation of the adaptive response. They also found that RFA dramatically increased circulating IL-6 at day 3 but this decreased to baseline by day 30, consistent with the supposed anti-tumour effect (Giardino et al. 2017). This study strongly supports the immunomodulatory effects of RFA in locally advanced pancreatic cancer. This study provides a promising platform and a need for more clinical trials to study the immunomodulatory effects of RFA in PDAC as a possible therapeutic intervention.

Furthermore, studies have reported the success of needle-guided ablative immunomodulation such as IRE (irreversible electroporation) to be effective in treating PDAC. Scheffer et al. report in a ten-patient study presenting with locally advanced pancreatic cancer (LAPC) who were treated with IRE, an upregulation of PD-1 observed on CD4^+^ and CD8^+^ T cells with a downregulation of Tregs in addition to a tumour antigen specific T cell response related to a better overall survival (Scheffer et al. 2019). These results suggest an immunogenic period after IRE which may be further leveraged by PD-1 inhibition. Although this was a small study, the findings are consistent and support the combination of IRE with therapeutic immune modulation. Pandit and colleagues analysed the peripheral blood samples of 11 patients with LAPC following IRE pre and post-surgery. They report a reduction in Treg populations following 3–5 days after treatment whereas a moderate increase was observed followed surgical intervention (Pandit et al. 2019). These findings suggest that IRE might alleviate immunosuppression in patients with LAPC and supports the notion for combination treatment. Zhao et al. show in a murine orthotopic pancreatic cancer model tumour infiltration by CD8^+^ cytotoxic T cells and a significant increase in survival by combination of IRE with anti-PD1 blockade. Additionally, they report a durable memory T cell response with a cure rate of 36–43% (Zhao et al. 2019).

In addition to IRE’s role in irreversibly permeabilizing the cell membrane and inducing apoptosis it also creates a reversible zone that could enhance drug delivery (Chen et al. 2019). As reported in an in vivo study by Bhutiani and colleagues, they show enhanced delivery of gemcitabine to the tumour by a possible change to the stroma, however the precise explanation for this remains unclear (Bhutiani et al. 2016). This method could also be customised to tumours negative for human equilibrative transporter 1 (hENT1) as hENT1 is a protein involved in the uptake and intracellular metabolism of gemcitabine.

## How do we know what therapy combinations will be most effective and in which patients?

Data from Cancer Research indicate that 403 immunotherapy combination trials opened globally in the first 6 months of 2017 alone with over 1100 in progress (Schmidt 2017), so much needed clinical outcome data are likely to be forthcoming over the next few years. It is highly unlikely that one treatment will be suitable for all however, and therefore we are going to need to develop biomarkers specific for individual treatments and combinations. A further interesting issue to consider is that early trials are all performed on heavily pre-treated patients with advanced disease. It may be that immunotherapy and checkpoint inhibition in particular produce better results in earlier disease as a result of immune editing of the tumour at later stages. A primed immune system will find it easier to target micrometastases of PDAC rather than bulky tumour masses containing dense desmoplasia. There is good data in mice models of breast cancer indicating superior therapeutic power of neoadjuvant immunotherapy compared with adjuvant, largely attributed to an elevated and sustained peripheral tumour-specific immune response (Liu et al. 2016). Recent work using multiple models of spontaneous metastases has demonstrated the efficacy of extended release local immunotherapy in the perioperative setting to prevent tumour recurrence as well as eliminate metastases (Park et al. 2018). Should we therefore turn our attention to patients who are receiving neoadjuvant treatment for borderline or locally advanced disease or even those that are operable? Treatments such as RFA already have proven oncological efficacy and using modern endoscopic ultrasonography, this can be easily and safely delivered in the neoadjuvant setting to turn the “cold” pancreatic tumour into one that is more easily recognised by the immune system and combined with checkpoint inhibition and other therapies described here to effectively in situ vaccinate the patient against any recurrence based on the characteristics of their individual disease.

### Patient sub-stratification

The immune landscape of pancreatic cancer has emerged as an important prognostic feature for future targeted therapies. A pan cancer analysis of more than 10, 000 tumours have reported 6 immune subtypes to exist (Thorsson et al. 2018). These subtypes could be classified according to their microenvironment, genetic and prognostic features. This underlines the importance of understanding and appreciating the spectrum of tumour-immune interactions for paving the way for combinatorial immunotherapy. Studies analysing the molecular characteristics of various PDAC subtypes have evolved through the past years. Originally these subtypes were identified as the classical, quasi-mesenchymal and exocrine types (Collisson et al. 2011) which overlap with Bailey et al.’s published subtypes (Bailey et al. 2016). Using a different approach, Poudel et al. report previously unidentified heterogeneity in pancreatic cancers and report immune cell-type differences are prevalent indicating patient sub-stratification importance for immunotherapy (Poudel et al. 2017). Further analysis revealed an enrichment of genes in the inflammatory subtype in co-stimulation of MHC class I, type I interferon (INF), APC (CoS-APC) and macrophages (Poudel et al. 2017). Therefore, heterogeneity across these subtypes is influenced by the immune microenvironment (Poudel et al. 2017).

In depth, molecular analysis of these subtypes have revealed definitive gene signatures which may be primed to target the most beneficial treatment for the patient (Collisson et al. 2011). The response to different chemotherapy drugs has differed between these subtypes with the classical subtype being a good responder to gemcitabine in comparison to the quasi-mesenchymal subtype (Collisson et al. 2011). In addition, Puleo et al. in redefining the subtypes, report hENT1, a marker of gemcitabine sensitivity to be significantly enriched in the classical subtypes (Puleo et al. 2018) thus patients with this subtype would benefit from gemcitabine treatment. These insights into the different landscapes of PDAC will no doubt aid in better sub stratification for efficient clinical outcomes as understanding the biology involved will help to understand the management of PDAC. Overall, the different immune, stromal and cancer-associated fibroblast subtypes have already provided us with a plethora of players involved in PDAC. Together with immunotherapy, this may provide the power calculation needed for enhancing overall survival of this recalcitrant disease.

## Conclusion

A final consideration is that whilst checkpoint inhibition is the main immunotherapy licensed for routine clinical practice of solid tumours at present, in the future it will likely be only one part of the ultimate armamentarium of immunotherapy, with thousands of new treatments under development. Due to the known heterogeneity of treatment responses in PDAC, we are most certainly heading towards a future of individualised therapy plans based on specific genetic germline tumour characteristics and guided by a battery of “-omic” biomarkers, and this is likely to be what is needed to break the deadlock in progress on pancreatic cancer survival. To leverage the full potential of checkpoint blockade in PDAC at present, we need to evaluate the neoadjuvant group and think outside the box when it comes to novel combination treatments.
